# Different topological patterns in structural covariance networks between high and low delay discounters

**DOI:** 10.3389/fpsyg.2023.1210652

**Published:** 2023-08-30

**Authors:** Wi Hoon Jung, Euitae Kim

**Affiliations:** ^1^Department of Psychology, Gachon University, Seongnam, Republic of Korea; ^2^Department of Psychiatry, Seoul National University College of Medicine, Seoul, Republic of Korea; ^3^Department of Brain and Cognitive Sciences, Seoul National University College of Natural Sciences, Seoul, Republic of Korea

**Keywords:** delay discounting, graph theoretical analysis, impulsivity, intertemporal choice task, structural covariance network

## Abstract

**Introduction:**

People prefer immediate over future rewards because they discount the latter’s value (a phenomenon termed “delay discounting,” used as an index of impulsivity). However, little is known about how the preferences are implemented in brain in terms of the coordinated pattern of large-scale structural brain networks.

**Methods:**

To examine this question, we classified high discounting group (HDG) and low discounting group (LDG) in young adults by assessing their propensity for intertemporal choice. We compared global and regional topological properties in gray matter volume-based structural covariance networks between two groups using graph theoretical analysis.

**Results:**

HDG had less clustering coefficient and characteristic path length over the wide sparsity range than LDG, indicating low network segregation and high integration. In addition, the degree of small-worldness was more significant in HDG. Locally, HDG showed less betweenness centrality (BC) in the parahippocampal gyrus and amygdala than LDG.

**Discussion:**

These findings suggest the involvement of structural covariance network topology on impulsive choice, measured by delay discounting, and extend our understanding of how impulsive choice is associated with brain morphological features.

## Introduction

Impulsivity is a complex and multidimensional concept that refers to the tendency to act without considering the potential negative consequences or long-term effects of those actions and inability to inhibit inappropriate behaviors (Patton et al., 1995; Whiteside and Lynam, 2001; Reynolds et al., 2006). Because of its multifaceted nature, impulsivity plays a significant role in numerous psychiatric disorders, such as personality disorders, behavioral disorders, substance use disorders, and bipolar disorder (Moeller et al., 2001; Berlin and Hollander, 2014). In these conditions, individuals exhibit different impulsive behaviors that can have detrimental effects on their lives and well-being.

Impulsivity can be assessed using self-report questionnaires and by behavioral tasks. One commonly used self-report questionnaire is the Barratt Impulsiveness Scale. Factor analysis of this scale has indicated a three-factor structure, which includes attentional impulsiveness (reduced attention), motor impulsiveness (increased motor activation), and non-planning impulsiveness (decreased planning) (Patton et al., 1995). In addition to self-report measures, behavioral tasks like the intertemporal choice task, also known as a delay discounting (DD) task, are commonly used to assess impulsivity, particularly impulsive choice. This task involves making choices between smaller-but-immediate and larger-but-delayed rewards (Kable and Glimcher, 2007). Because impulsivity and reward-seeking are closely linked (Donohew et al., 2000; Whiteside and Lynam, 2001), impulsive individuals are more prone to engaging in reward-seeking behaviors without considering potential long-term effects. Thus, the immediate gratification associated with reward-seeking behaviors can be appealing to impulsive individuals, leading them to prioritize short-term gains over long-term benefits. The DD task measures such people’s preference (called the DD rate) for smaller-but-immediate rewards over larger-but-delayed rewards, and the DD rate is used as behavior index of impulsivity (Kable and Glimcher, 2007; Levitt et al., 2020).

Understanding individual differences in the DD rate is important because it is associated with various real-life consequences (Chabris et al., 2008; Madden and Bickel, 2010; Levitt et al., 2020). For instance, higher discounting rates are associated with drug use (Kirby and Petry, 2004) and obesity (Fields et al., 2013) and are observed in a variety of psychiatric disorders (e.g., addiction, attention-deficit hyperactivity disorder, bipolar disorder, and schizophrenia; Ahn et al., 2011; Story et al., 2016). By contrast, low discounting rates are linked to academic achievement (Kirby et al., 2005). Therefore, investigating the neural mechanism underlying DD using a neuroeconomic approach may predict future impulsive and addictive behaviors and a potential target for effective interventions to reduce such behaviors and symptoms.

Over the last two decades, functional neuroimaging studies have identified multiple brain areas activated in the intertemporal choice (Kable and Glimcher, 2009; Peters and Büchel, 2009; Chen et al., 2019a). These brain areas include the ventral striatum, ventromedial prefrontal cortex, and posterior cingulate cortex, where neural activity encodes the subjective value (*SV*) of given options during the task (Kable and Glimcher, 2007; Bartra et al., 2013; Clithero and Rangel, 2014). In addition, activity in the lateral prefrontal cortex and medial temporal areas involved in choosing options based on *SV* and in imaging future outcomes were also observed (Ballard and Knutson, 2009; Kable and Glimcher, 2009; Peters and Büchel, 2009; Figner et al., 2010; Lebreton et al., 2013). Consistent with these neurofunctional findings, anatomical neuroimaging studies have shown the association between DD and brain morphology (e.g., gray matter [GM] volume and cortical thickness) in these abovementioned areas, such as the striatum, medial prefrontal and temporal regions, and lateral prefrontal cortex (Bjork et al., 2009; Dombrovski et al., 2012; Cho et al., 2013; Lebreton et al., 2013; Wang et al., 2016; Owens et al., 2017). Therefore, it is suggested that functional and anatomical differences in these areas are associated with individual differences in DD.

Covariance in GM morphology between different brain areas may be a powerful tool for inferring large-scale structural brain networks. Structural covariance patterns between different regions are similar to functional connectivity (Zielinski et al., 2010; Yun et al., 2020). This similarity suggests that coordinated covariance in brain morphology reflects developmental coordination between areas (Alexander-Bloch et al., 2013). More recently, using graph theoretical network analysis (which characterizes the network’s topological properties) investigations have reported that the topology of structural covariance networks follows small-world network properties (He et al., 2008; Zhang et al., 2012). These networks are characterized by high clustering (local segregation) and low path length (global integration) among nodes (i.e., brain areas) in the network (Watts and Strogatz, 1998).

Furthermore, studies have demonstrated disrupted small-world topology in the structural covariance networks in various neurologic or psychiatric disorders, including Alzheimer’s disease (He et al., 2008), schizophrenia (Zhang et al., 2012), and OCD (Yun et al., 2020). Based on these findings, graph theoretical analysis in conjunction with structural covariance networks may provide novel insights into neural mechanisms underlying DD (i.e., impulsive behavior) at the network level. No studies so far have investigated differences in the topology of the structural covariance networks based on GM morphology according to the discounting rate, while a few studies have investigated the relationship between DD and the topological properties of networks generated from functional connectivity or structural connectivity derived from diffusion tensor imaging (DTI) (Chen et al., 2018; Wang et al., 2021a).

Therefore, we investigated differences in the topological organization of GM volume-based structural covariance networks between two groups discriminated in terms of high and low discounting rates in the intertemporal choice using graph theoretical analysis. Among the coordinated patterns of brain morphology (i.e., graph theoretical metrics estimated from GM volume-based structural covariance networks), we especially expected that there would be differences in small-world parameters that reflect a balance between information segregation and integration between brain regions, and in nodal betweenness centrality (BC) that indicates the relative importance of a node in the network. To address this issue, several global network parameters (small-world parameters including clustering coefficient, characteristic path length, and small-worldness) were computed to quantify small-world structure in the network (Watts and Strogatz, 1998). In addition, we calculated the BC as a regional (nodal) network parameter to quantify the relative importance for each of nodes and is used to identify a hub that acts as a bridge between nodes in the network (Freeman, 1977; He et al., 2008).

## Methods

### Participants

Participants involved in the current study were recruited as part of the Psychological and Neural Mechanisms for Predicting Academic Achievement (PNMPAA) study. For the PNMPAA study, participants were asked to fill out a series of surveys (topics included their achievement goals, motivation, time perspectives, and personality traits), performed choice behavior tasks, and underwent brain scans. During some cognitive tasks, the scanning session consisted of high-resolution T1-weighted anatomical MRI, resting-state fMRI, DTI, and fMRI. In this study, we used T1 data to examine whether there are any differences in topological properties of GM-based structural covariance networks between the high (HDG) and low discounting groups (LDG). All participants in the present study were young, healthy adults who had normal or corrected-to-normal vision and no significant medical illness. They gave written informed consent before participation. All study procedures were approved by the Institutional Review Board of Gachon University (IRB number: 1044396-202203-HR-056-01). All methods were performed in accordance with the relevant guidelines and regulations.

Of the entire cohort (*N* = 115) collected to date, 73 completed both the DD task and brain scans. Two individuals out of 73 were excluded because of (i) data missing (*n* = 1) and (ii) low data quality (*n* = 1). We split participants at the median *k* value into two groups of high (*n* = 35) and low (*n* = 35) discounters after excluding one individual who scored the median value. Therefore, 70 participants were used in the final analysis (Table 1).

**Table 1:** Demographic and behavioral data.

Variables	High discounters	Low discounters	*t* or *χ^2^*	*p*
Age (years)	21.943 ± 2.920	22.057 ± 2.555	−0.174	0.862
Sex (male/female)	21/14	18/17	0.521	0.470
Education (years)	14.800 ± 1.132	15.286 ± 1.447	−1.564	0.122
Discounting rate (*k*)	0.029 ± 0.022	0.006 ± 0.003	6.193	p < 0.001
Total intracranial volume (mm^3^)	1584.088 ± 163.212	1546.765 ± 138.138	1.033	0.305

Values are presented as mean ± standard deviation.

### Task

**Figure 1:**
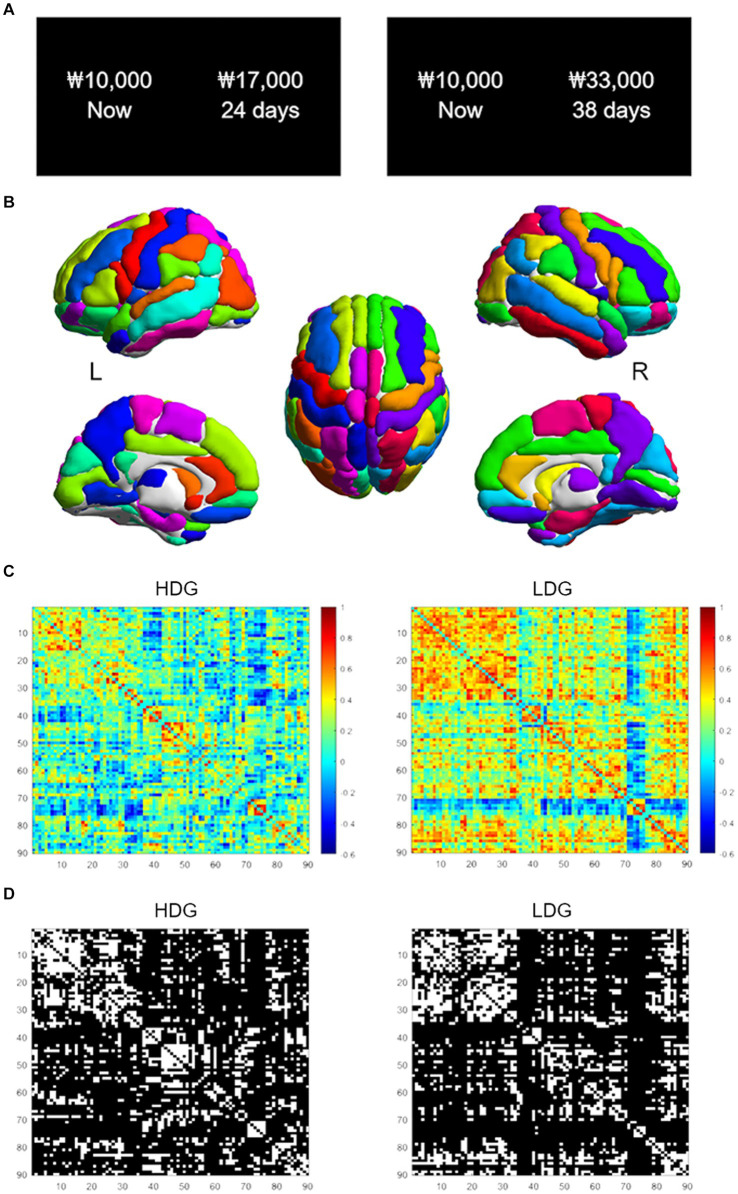
Intertemporal choice task and structural covariance networks. **(A)** Examples of task trials. Participants chose between a smaller-immediate reward (10,000 won now) and a larger-delayed reward (17,000 won in 24 days). **(B)** The AAL atlas used to segment the brain into 90 nodes. **(C)** The 90 by 90 correlation matrices for the HDG (left column) and LDG (right column). The color bar indicates the Pearson correlation coefficient on the matrices. **(D)** Binarized matrices thresholded at 0.25 sparsity. The correlation matrices of **(C)** were thresholded into the binarized matrices with a wide range of sparsity (0.25–0.53, with an interval of 0.01). HDG, high discounting group; LDG, low discounting group.


The task displayed monetary amounts in KRW (₩). Participants were asked to make a series of 120 choices between a smaller-immediate reward, fixed at ₩10,000 now for all trials, and a variable larger-delayed reward (Figure 1A). The magnitude of the larger-delayed reward ranged from ₩11,000 to ₩48,000, and the delay varied from 2 to 180 days. Discounting rates (*k*) were estimated by fitting logistic regressions that assume an individual’s decisions are a stochastic function of the difference in *SV* between given two options. In other words, a logistic choice rule was used to compute the probability of choosing options as a function of the difference in the *SV* of two choice options on each trial as follows:

$$P_{1}=\frac{1}{1+e^{-\beta \left(SV1-SV2\right)}},P_{2}=1-P_{1}$$

where *P*_1_ refers to the probability that the participant chose the delayed option, and *P*_2_ refers to the probability that the participant chose the immediate option. *SV1* and *SV2* refer to the participant’s estimated subjective value of the delayed and the immediate options, respectively. *β* was used as a scaling factor and was fitted for each subject. Keeping with standard behavioral findings (Mazur, 1987; Kable and Glimcher, 2007), we assume that *SV* is a hyperbolic function of the reward amount (*A*) and delay (*D*): *SV* = *A*/(1 + *kD*), where *k* is the participant’s discount rate. Larger *k* values represent a greater degree of discounting future rewards. The *k* was log-transformed to normalize the distribution before statistical analyses.

### Image acquisition and preprocessing

All imaging data were acquired on a 3 T Trio MRI scanner (Siemens, Erlangen, Germany). High-resolution T1-weighted anatomical images were obtained using a 3D magnetization-prepared rapid-gradient echo (MPRAGE) sequence (repetition time [TR] = 1,900 ms, echo time [TE] = 2.52 ms, flip angle [FA] = 9°, voxel size = 1.0 × 1.0 × 1.0 mm^3^, 192 sagittal slices). Other image parameters unrelated to the present study are not described here.

Image preprocessing was conducted using the Computational Anatomy Toolbox (CAT^1^) for SPM12^2^ with default options. First, all structural images were segmented into GM, white matter (WM), and cerebrospinal fluid images. Then, high-dimension DARTEL normalization was applied to normalize and modulate the GM images (voxel size = 1.5 × 1.5 × 1.5 mm^3^).

### Structural covariance network construction

The Automated Anatomical Labeling (AAL; Tzourio-Mazoyer et al., 2002) atlas was used to segment the brain into 90 cortical and subcortical regions (45 per hemisphere; Figure 1B) as nodes in the network. Regional GM volumes of each area were extracted during the CAT preprocessing. First, we regressed age, sex, education, and total intracranial volume (TIV) effects on GM volumes by linear regression analysis (He et al., 2007). We then performed Pearson correlations between the corrected GM volumes to construct a 90 × 90 correlation matrix for each group (Figure 1C). Only positive correlations of the matrix were considered as edges (i.e., connections). Negative correlations were assigned a zero value before subsequent network analysis (Zhang et al., 2019). Then, the correlation matrix was binarized with a fixed sparsity threshold to ensure that both groups had the same number of edges on the binarized network (Figure 1D). As there is not a gold standard to select a single threshold, we used a wide range of sparsity thresholds (0.25–0.53, with an interval of 0.01). The sparsity threshold range was selected to allow for a small-world regime in the brain networks of both groups; that is, the small-worldness (σ) of the threshold networks was greater than 1 (Watts and Strogatz, 1998; Zhang et al., 2012; Jung et al., 2016).

### Network topological properties

We estimated both global and regional topological properties in the structural covariance networks using the Brain Connectivity Toolbox (BCT, Rubinov and Sporns, 2010)^3^ with MATLAB R2021b. Small-world parameters (including clustering coefficient, characteristic path length, and small-worldness) were computed to characterize global topological properties [refer to Rubinov and Sporns (2010) for a detailed equation for each parameter]. Briefly, the clustering coefficient of a node is the ratio of the number of existing edges between direct neighbors of the node to the number of all possible edges between them. The network clustering coefficient is defined as the clustering coefficient average across all network nodes, reflecting its segregation. The shortest path length between two nodes is the minimum number of edges included in the path connecting these two nodes. The characteristic path length of a network is defined as the average shortest path length between all node pairs in the network, which measures network integration. Small-worldness is defined as the ratio of normalized clustering coefficient to normalized characteristic path length. Therefore, before computing the ratio, we normalized by comparing the clustering coefficient and characteristic path length to the corresponding mean values of 100 matched random networks.

BC as the regional network parameter was estimated to characterize regional topological organization at a sparsity threshold of 25%. This sparsity ensured that all nodes were included in the network for both groups while minimizing the number of false-positive connections (He et al., 2008). BC is the fraction of all shortest paths in the network that pass through a given node. The BC of a node *i* on a given graph *G* with *N* nodes is calculated through the following formula (Freeman, 1977; Jung et al., 2013; Gharahi et al., 2023):


$$BC\left(i\right)=\underset{j\neq i\neq k\in G}{\sum }\frac{\delta_{jk}\left(i\right)}{\delta_{jk}}$$

Where δ *_jk_* is the total number of shortest paths from a node *j* to a node *k*, and δ *_jk_* (*i*) is the number of those paths that pass through a node *i* within graph *G.* The BC value reflects the influence of a node on the information flow between other nodes in the network. Before group comparison, the BC was normalized by the average BC of the network. Nodes having greater than one standard deviation above the average BC across all nodes were considered hubs for each group (Bassett et al., 2008; Jung et al., 2016). Finally, the hub locations were qualitatively compared across groups.

### Statistical analysis

A nonparametric permutation test (1,000 repetitions) was performed to determine the statistical significance of differences in the network topological properties between groups (Bullmore et al., 1999; He et al., 2008). At each permutation, the corrected GM volumes of all participants were randomly reassigned to one of two new groups. The correlation matrix for each randomized group was recomputed and binarized over the range of defined sparsity thresholds. The topological network properties were estimated for each thresholded network. In addition, their intergroup differences were computed to create a permutation distribution of differences under the null hypothesis. The significance level was set at *p* < 0.05 for group differences in global and regional topological properties. Following previous studies (Lynall et al., 2010; Hong et al., 2013; Jung et al., 2016), for the regional parameter (i.e., BC), we also applied a threshold of *p* < 0.011 (=1/90, 90 is the number of nodes), which is a less stringent false positive correction based on the number of nodes. BrainNet Viewer was employed for network visualization (Xia et al., 2013).

## Results

### Demographic and behavioral data

HDG, compared with LDG, showed significantly greater discounting rates (*t* = 6.193, *df* = 68, *p* < 0.001; Table 1). However, other variables, including age, sex, education, and TIV, were not significantly different between the two groups (*ps* > 0.05).

### Global network analysis

**Figure 2:**
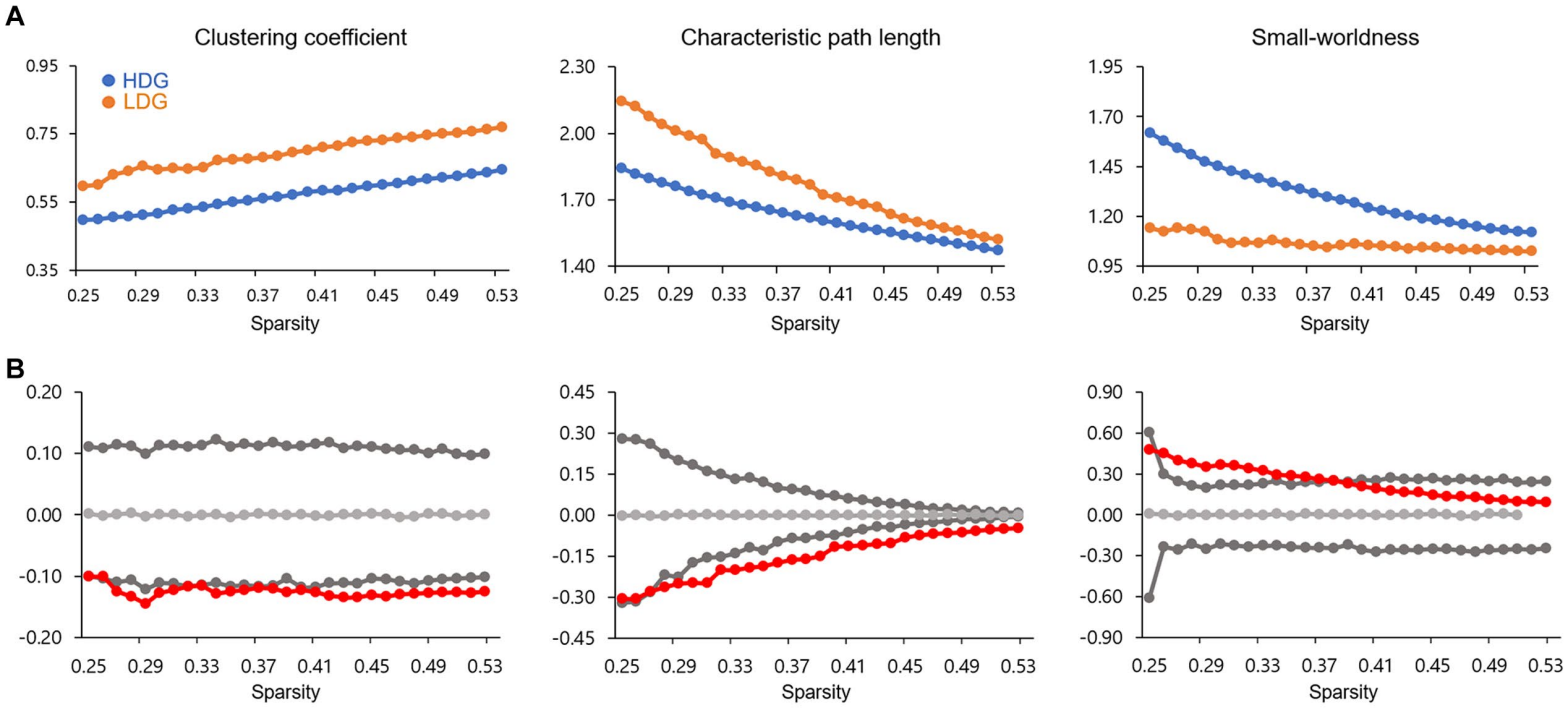
Global network topological properties of the structural covariance network. **(A)** Changes in small-world parameters (including clustering coefficient, characteristic path length, and small-worldness) in HDG (blue circles) and LDG (orange circles) as a function of network sparsity. **(B)** Differences (red circles) in global network properties between two groups. The gray lines represent the mean values (light gray), and 95% confidence intervals (dark gray) of the between-group differences obtained 1,000 permutation tests at each sparsity threshold. The red circles lying outside of the confidence intervals indicate the sparsity where the difference is significant at *p* < 0.05. The positive values indicate HDG > LDG, and negative values indicate HDG < LDG.

The GM-based structural covariance networks for both groups followed a small-world architecture across the defined sparsity range. This pattern was evidenced by small-worldness (σ) > 1 (Figure 2A) as well as normalized clustering coefficient > 1 and normalized characteristic path length ≈ 1, generated by comparing with random networks (Watts and Strogatz, 1998; Humphries et al., 2006).

As shown in Figure 2B, significant differences in clustering coefficient and characteristic path length between the two groups were detected with a wide range of sparsity thresholds (0.27 < sparsity <0.53), showing that HDG had fewer values in these two global parameters, relative to LDG. In addition, significant differences in small-worldness were also observed at 0.26 < sparsity <0.38, showing that HDG, relative to LDG, showed greater small-worldness.

### Regional network analysis

**Figure 3:**
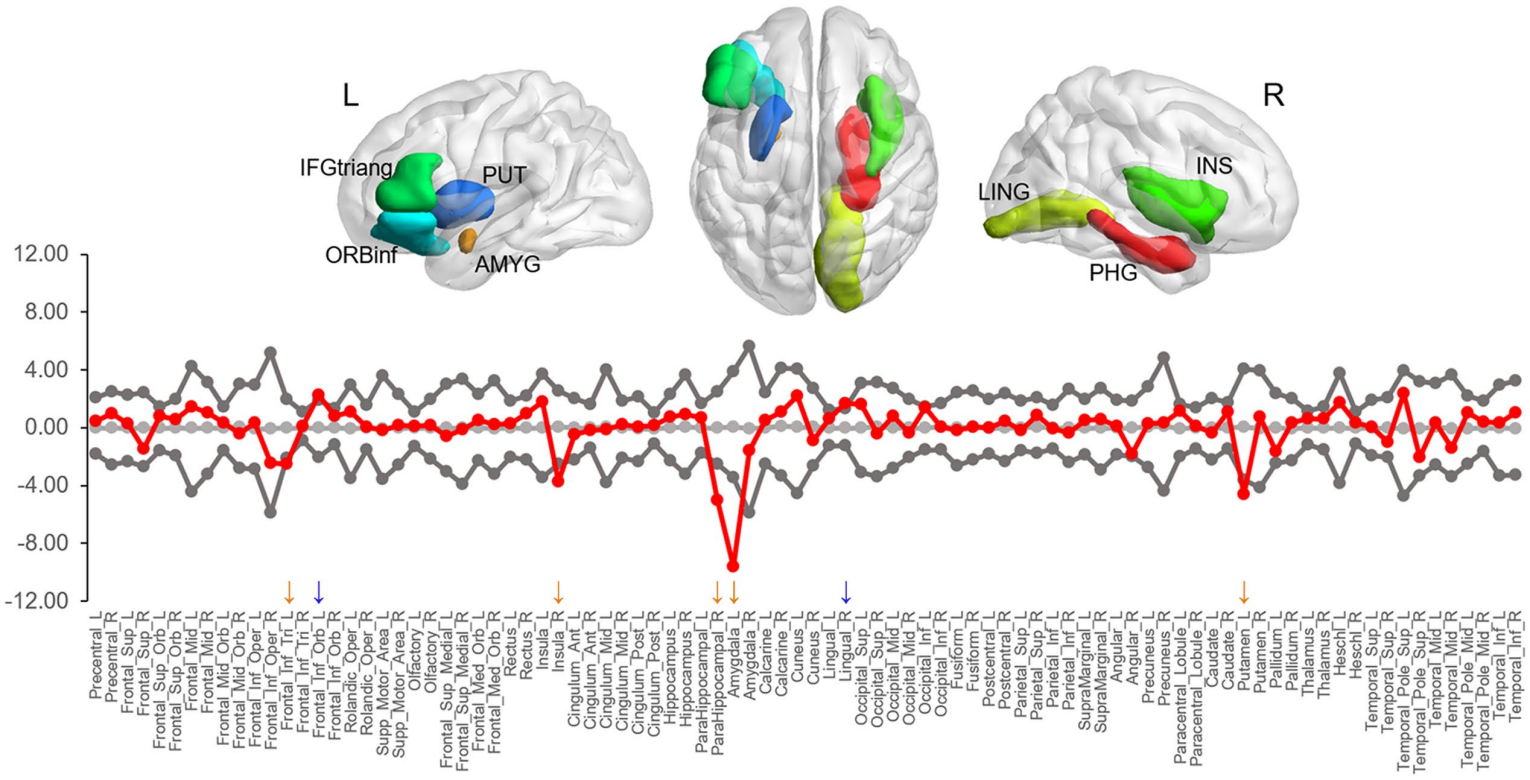
The difference in nodal betweenness centrality between two groups. Regions showing significant differences are rendered on a brain surface. The graph shows the differences (red circles) in normalized betweenness centrality for each node between two groups. The gray circles and lines represent the mean values and 95% confidence intervals of the between-group differences obtained from 1,000 permutation tests. The red circles lying outside of the confidence intervals indicate the sparsity where the difference is significant at *p* < 0.05. The positive values (blue arrows) indicate HDG > LDG, and negative values (orange arrows) indicate HDG < LDG. L, left; R, right; IFGtriang, triangular inferior frontal gyrus; PUT, putamen; ORBinf, orbital inferior frontal gyrus; AMYG, amygdala; LING, lingual gyrus; PHG, parahippocampal gyrus; INS, insula.

Group differences in the BC were observed in seven areas (at *ps* < 0.05), including the right parahippocampal gyrus (*p* = 0.001), left amygdala (*p* = 0.005), right lingual gyrus (*p* = 0.020), right insula (*p* = 0.022), left triangular inferior frontal gyrus (IFGtriang, *p* = 0.024), left orbital inferior frontal gyrus (ORBinf, *p* = 0.024), and left putamen (*p* = 0.033) (Figure 3). Compared to LDG, HDG showed greater BC in the lingual gyrus and ORBinf but less BC in the IFGtriang, parahippocampal gyrus, insula, amygdala, and putamen. However, of all these areas, only two limbic areas (the left amygdala and right parahippocampal gyrus) showing less BC in HDG survived when applying a false positive correction.

**Figure 4:**
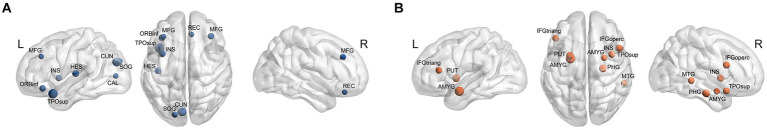
Hubs in each group. **(A)** Hubs in the HDG (blue circles) were located in 10 cortical areas (including right middle frontal gyrus [MFG], right rectus [REC], left MFG, left orbital inferior frontal gyrus [ORBinf], left insula [INS], left superior temporal pole [TPOsup], left Heschl gyrus [HES], left cuneus [CUN], left superior occipital gyrus [SOG], and left calcarine [CAL]). **(B)** Hubs in the LDG (orange circles) were 6 cortical (right opercular inferior frontal gyrus [IFGoperc], right INS, right TPOsup, right middle temporal gyrus [MTG], right parahippocampal gyrus [PHG], and left triangular inferior frontal gyrus [IFGtriang]) and 3 subcortical (right amygdala [AMYG], left AMYG, and left putamen [PUT]) areas.

A different number and distribution of network hubs were observed between the two groups (Figure 4). Especially, 10 regions (all cortical; left superior temporal pole, left cuneus, left Heschl gyrus, left ORBinf, left insula, right middle frontal gyrus, left superior occipital gyrus, left calcarine, right rectus, and left middle frontal gyrus) were identified as hubs in the HDG. Nine hubs (six cortical and three subcortical; right insula, right opercular inferior frontal gyrus, right middle temporal gyrus, right superior temporal pole, right parahippocampal gyrus, left IFGtriang, left and right amygdala, left putamen) were identified in the LDG. No regions were common to both groups.

## Discussion

In the present study, we investigated whether there were differences in the coordinated patterns (i.e., topological properties from graph theoretical analysis) of structural covariance networks between HDG and LDG. Earlier studies showed brain areas associated with DD in terms of brain morphology, suggesting neuroanatomical correlates of DD (Bjork et al., 2009; Dombrovski et al., 2012; Cho et al., 2013; Lebreton et al., 2013; Wang et al., 2016; Owens et al., 2017; Pehlivanova et al., 2018). The present study extends these findings by being the first to examine the involvement of topological features of the structural covariance networks generated based on brain morphology (i.e., GM volume) on impulsive choice, measured by DD, at group level. We found significant differences in global network topology, especially small-world parameters, between HDG and LDG. HDG showed fewer values in both clustering coefficient and characteristic path length but greater small-worldness. We also observed differences in the regional network topology, particularly BC, between groups. HDG had lower BC in the limbic region, particularly the parahippocampal gyrus and amygdala. Together with earlier findings showing an association between DD and regional GM volumes, these findings provide evidence for the involvement of brain morphology in DD at group level. Our findings further suggest that the topological characteristics of brain morphology-based structural covariance networks may play a central role in impulsive choice.

To construct brain networks in the current study, we used structural covariance that is an indicator of individual differences in brain volumetry within a group. Structural covariance networks reflect the degree to which the morphology (in this case, regional GM volume) of brain regions covaries with other regions within a given group. That is, a group with high (low) structural covariance would have high (low) correlations between regional GM volumes across individuals in that group. Therefore, the current results we found argue differences in the structural covariance patterns between groups divided by discounting rates (the index of impulsive choice). Though the precise neurobiological and development mechanisms behind structural covariance patterns remains unclear, structural covariance networks share several common topological features with functional brain networks, such as small-world topology and hubs (Achard et al., 2006; He et al., 2007), and previous studies have suggested environment-related structural changes (Maguire et al., 2000) or mutually trophic effects (Ferrer et al., 1995) on structural covariance patterns. Some studies have also demonstrated disrupted topological features in the structural covariance networks in various neurologic or psychiatric disorders, including Alzheimer’s disease (He et al., 2008), schizophrenia (Zhang et al., 2012), and OCD (Yun et al., 2020).

A small-world network is characterized by a high clustering coefficient and a short average shortest path length (i.e., a short characteristic path length), which means high segregation and integration of the network, respectively (Watts and Strogatz, 1998). Therefore, the small-world network is suggested to support efficient information processing. The extent to which a given network displays small-world structure (quantified as small-worldness) can be evaluated by considering the balance between segregation and integration (Watts and Strogatz, 1998). In other words, small-worldness is the ratio of the clustering coefficient [numerator] to the characteristic path length [denominator], normalized by comparing to corresponding values of random networks. If the small-worldness of a given network is greater than 1, the network is deemed to be a ‘small-world.’ In the current study, we observed small-world structures (small-worldness >1) particularly in the structural covariance networks over the wide range of sparsity thresholds, consistent with previous studies (He et al., 2008; Zhang et al., 2019). We also showed that HDG, compared with LDG, had fewer levels of both clustering coefficient and characteristic path length. Therefore, the results are interpreted as less segregation and higher integration in HDG. Small-worldness was higher in HDG compared with LDG. Though HDG showed a greater small-worldness value, we would urge caution in interpreting this result because HDG and LDG are young, healthy adults who have no serious clinical problems. Considering the equation to calculate the small-worldness presented above, higher small-worldness can come out if the characteristic path length [denominator] is relatively smaller than the clustering coefficient [numerator]. Therefore, higher small-worldness in HDG may be due to even less characteristic path length than LDG. Indeed, the characteristic path length difference between the two groups was much more significant than the difference in the clustering coefficient (see Figure 2).

Previous studies have demonstrated small-world structure in structural and functional brain networks, derived from DTI tractography and functional connectivity, respectively (Gong et al., 2009; Lo et al., 2010; Jung et al., 2013, 2016). Many studies have revealed differences in the topological properties of these structural or functional brain networks not only in a variety of clinical conditions, such as Alzheimer’s disease, OCD, and schizophrenia (Lo et al., 2010; Shin et al., 2014; Jiang et al., 2022) but also between groups divided according to experience or skills even in healthy adults (e.g., board game experts versus novices; Jung et al., 2013). However, a few studies have explicitly investigated brain network topology involvement in DD (Chen et al., 2019b; Wang et al., 2021a,b). They showed the association between individual differences in DD and global network topological properties, such as small-world parameters. For example, using DTI and resting-state fMRI data, Chen et al. (2019a,b) investigated the association between individual DD and global and regional network properties estimated from structural and functional brain networks. They found that high discounters had decreased small-world parameters, including normalized clustering coefficient and small-worldness, in structural and functional brain networks. However, they did not observe any significant association with regional network properties. More recently, Wang et al. (2021a) applied the representational connectivity analysis (RCA) approach to generate functional brain networks corresponding to future rewards’ amount and delay time during the DD task. In addition, they investigated the relationship between DD and topological parameters of these two networks. They found that global network topology (global efficiency) in the delay-related network was inversely associated with DD. Discrepancies between previous findings and current findings may stem from differences in image modality and analysis method used to generate the brain network and node definition.

In the present study, we found BC differences in several regions between the two groups. Among them, the amygdala and parahippocampal gyrus remained after multiple comparison corrections, suggesting that the local network topology of the limbic areas may play a more central role in DD. Previous functional and structural studies have reported the involvement of the medial temporal regions including the amygdala and parahippocampal gyrus in DD, suggesting the role of these areas in impulsive choice. Dysfunction in amygdala and parahippocampal gyrus may each lead to a preference for immediate rewards associated with positive emotions or memories, even at long-term disadvantages. The amygdala is a key brain region for reward and emotional processing (Hommer et al., 2003; Yang et al., 2020). The amygdala responds to the salience of stimulus and prepares adaptive behaviors for changing environmental conditions (Cunningham and Brosch, 2012). In the context of impulsive choice, the amygdala is associated with the evaluation of immediate stimuli and their emotional significance. For instance, amygdala activation in humans is correlated with reward magnitude for immediate over delayed rewards (Ludwig et al., 2015). Aberrant activation and lesions in the amygdala are related to preferences for immediate rewards in rodents (Winstanley et al., 2004; Churchwell et al., 2009). Given the abovementioned findings, when faced with a choice that entails immediate reward, the amygdala can influence impulsive decision-making by biasing individuals toward choosing options that provide immediate emotional satisfaction, even at long-term disadvantages. The parahippocampal gyrus, a cortical region surrounding the hippocampus, plays a vital role in memory (Squire and Zola-Morgan, 1991) and visuospatial processing (Aguirre et al., 1996). In the context of impulsive choice, the parahippocampal gyrus may play a role in evaluating the significance of available options based on the context in which they are presented. Activity in the parahippocampal gyrus can predict an individual’s DD (Chen et al., 2019b). Recently, Wang et al. (2021b) found that less all-range non-hub resting-state functional connectivity (also called degree centrality, which measures the sum of all the connections between a given voxel and all of the other voxels) in the parahippocampus was associated with high DD. Considering that the hippocampus and parahippocampal gyrus play a key role in imagining novel experiences (e.g., future thinking; Schacter et al., 2007, 2008), it is suggested that these areas may contribute to evaluating future rewards through mental simulation, that is a process of prospection (Johnson et al., 2007; Luhmann et al., 2008; Peters and Büchel, 2009). Given the abovementioned findings, the parahippocampal gyrus can influence impulsive decision-making by weighing the significance of the choices in relation to stored memories or imagined futures.

Some study limitations should be addressed. First, this study included only young, healthy adults. It is thus necessary to see if results from people with impulsive disorders are similar to the current findings. Second, as there are no individual networks but the group-level networks to estimate structural covariances, we could not examine the association between individual network topology parameters and DD. Third, considering previous results showing the association between structural and functional networks (Honey et al., 2007; Chen et al., 2019b), further studies combining networks generated from structural covariance and functional connectivity are needed to improve our understanding of the relationship between topological properties of structural covariance and functional brain networks and between these variables and DD. Additionally, future studies using DTI and functional MRI data will help clarify the association of topological properties of structural and functional connectivity with individual differences in DD.

In summary, this study applied a neuroeconomic approach to study the neural mechanisms underlying impulsivity, measured by the DD rate. To investigate DD-related differences in the coordinated patterns of large-scale structural brain networks, we compared global and regional topological properties of the GM volume-based structural covariance networks between HDG and LDG. Our findings provide evidence supporting the involvement of brain morphology in DD at group level and offer new insights into the network mechanisms underlying DD, showing differences in small-world parameters (less segregation and high integration) and BC (an importance role in limbic areas, including the parahippocampal gyrus and amygdala, on delayed gratification) between two groups. Future studies with patients with impulsive behaviors are warranted to explore this issue further.

## Data availability statement

The original contributions presented in the study are included in the article/supplementary material, further inquiries can be directed to the corresponding author.

## Ethics statement

The studies involving humans were approved by the Institutional Review Board of Gachon University. The studies were conducted in accordance with the local legislation and institutional requirements. The participants provided their written informed consent to participate in this study.

## Author contributions

WJ conceived the experiments, conducted the analysis, and wrote the manuscript. EK collected the data and wrote the manuscript. All authors contributed to the article and approved the submitted version.

## Funding

This work was supported by the National Research Foundation of Korea (NRF) grant funded by the Korea government (MSIT) (No. NRF-2022R1A2C1010704).

## Conflict of interest

The authors declare that the research was conducted in the absence of any commercial or financial relationships that could be construed as a potential conflict of interest.

## Publisher’s note

All claims expressed in this article are solely those of the authors and do not necessarily represent those of their affiliated organizations, or those of the publisher, the editors and the reviewers. Any product that may be evaluated in this article, or claim that may be made by its manufacturer, is not guaranteed or endorsed by the publisher.

## Footnotes

^1^ http://www.neuro.uni-jena.de/cat

^2^ http://www.fil.ion.ucl.ac.uk/spm/

^3^ https://sites.google.com/site/bctnet/
